# Variation within the Huntington's Disease Gene Influences Normal Brain Structure

**DOI:** 10.1371/journal.pone.0029809

**Published:** 2012-01-03

**Authors:** Mark Mühlau, Juliane Winkelmann, Dan Rujescu, Ina Giegling, Nikolaos Koutsouleris, Christian Gaser, Milan Arsic, Adolph Weindl, Maximilian Reiser, Eva M. Meisenzahl

**Affiliations:** 1 Department of Neurology, Technische Universität München, Munich, Germany; 2 Institute of Human Genetics, Technische Universität München, Munich, Germany; 3 Institute of Human Genetics, Helmholtz Zentrum Munich, German Research Center for Environmental Health, Munich, Germany; 4 Department of Psychiatry and Psychotherapy, Ludwig-Maximilians-University, Munich, Germany; 5 Department of Psychiatry, Friedrich-Schiller-University, Jena, Germany; 6 Department of Radiology, Ludwig-Maximilians-University, Munich, Germany; University Medical Center Groningen, Netherlands

## Abstract

Genetics of the variability of normal and diseased brain structure largely remains to be elucidated. Expansions of certain trinucleotide repeats cause neurodegenerative disorders of which Huntington's disease constitutes the most common example. Here, we test the hypothesis that variation within the IT15 gene on chromosome 4, whose expansion causes Huntington's disease, influences normal human brain structure. In 278 normal subjects, we determined CAG repeat length within the IT15 gene on chromosome 4 and analyzed high-resolution T1-weighted magnetic resonance images by the use of voxel-based morphometry. We found an increase of GM with increasing long CAG repeat and its interaction with age within the pallidum, which is involved in Huntington's disease. Our study demonstrates that a certain trinucleotide repeat influences normal brain structure in humans. This result may have important implications for the understanding of both the healthy and diseased brain.

## Introduction

Knowledge on the genetic determination of normal brain structure is limited. Trinucleotide repeats refer to a short DNA tract in which the same sequence of 3 base pairs is repeated several to many times in tandem. This genetic variation may influence normal brain structure in humans since it contributes to variation in behavioral traits in animals [1] and causes a number of neurodegenerative disorders in humans of which Huntington's disease (HD) constitutes the most common example. This autosomal-dominant disorder results from an expanded CAG trinucleotide repeat size (>35) within the first exon of the IT15 gene on chromosome 4 leading to a polyglutamin stretch. HD is characterized by the triad of involuntary movements, dementia, and behavioral disturbances [2]. Of note, symptoms become more severe and start earlier in life with increasing trinucleotide repeat size, and brain atrophy is pronounced within subcortical structures [2]. Here, we test the hypothesis that normal CAG repeat size influences brain structure in normal human subjects. In analogy to HD, we determined the influence of the longer CAG on subcortical structures and expected this effect to increase with age.

## Methods

### 1. Subjects

The MRI images of 278 normal subjects (females, 130; age range, 18–65 years; mean±standard deviation, 34±12; 25^th^/50^th^/75^th^ percentile, 25/30/41) that had served as healthy controls in several imaging studies (Department of Psychiatry and Psychotherapy, Ludwig-Maximilians University, Munich, Germany) were analyzed. For the respective studies, all subjects underwent a structured interview and neuropsychiatric evaluation. Exclusion criteria were a history of known neurological or mental illness including first degree relatives as well as previous head injury with loss of consciousness, corticosteroid medication in the medical history, previous alcohol or other substance abuse, and other mental illnesses including personality disorders. Beforehand, written informed consent was obtained after description of the respective study to the subjects. The studies were approved by the ethics committee of the medical faculty of the Ludwig-Maximilians-University Munich, and performed in accordance with the Declaration of Helsinki.

### 2. Magnetic resonance imaging

All brain images were acquired on the same 1.5T scanner (Magnetom Vision; Siemens, Erlangen, Germany) including a 3-dimensional magnetization prepared rapid acquisition gradient echo sequence (repetition time, 11.6 milliseconds; echo time, 4.9 milliseconds; total acquisition time, 9 minutes; number of acquisitions, 1; field of view, 230 mm; matrix, 512×512 pixels; and section thickness, 1.5 mm).

### 3. Voxel-based morphometry

For voxel-based morphometry (VBM), we used an extension of the SPM8 software (http://www.fil.ion.ucl.ac.uk/spm), the VBM8 toolbox (http://dbm.neuro.uni-jena.de/vbm8). Here, images are corrected for bias-field inhomogeneities, registered using linear (12-parameter affine) and nonlinear transformations, as well as tissue-classified into grey matter (GM), white matter (WM), and cerebrospinal fluid (CSF) within the same generative model [3]. The segmentation procedure is further refined by high dimensional warping also called “DARTEL” [4], by accounting for partial volume effects [5], by adaptive maximum a-posteriori estimations [6], and by a hidden Markov random field model [7]. The resulting GM images were modulated to account for volume changes resulting from the normalization process. We considered only non-linear volume changes so that further analyses did not have to account for differences in head size. Finally images were smoothed with a Gaussian kernel of 8 mm (FWHM).

### 4. Measurement of CAG repeat size

Fragment analysis was performed using the following primer pair CCTTCGAGTCCCTCAAGTCCTT (forward), GGTGGCGGCTGTTGCTGCTGC (reverse), which do not amplify the CCG-repeat adjacent to the CAG-repeat and analyzed on an ABI 3730 sequencer using LIZ-500 (ABI) as a standard. Analysis was performed using GeneMapper v3.5 [8].

### 5. Statistical analysis

For each subject, determination of the CAG repeat size yielded 2 values. In analogy to HD, we expected the longer CAG repeat to be primarily efficacious with regard to brain structure and even more so with increasing age. We used a voxel-wise general linear model (GLM) as implemented in SPM8. The main effect of a variable corresponds to a linear positive or negative relationship with GM. To estimate the main effect of a variable, a contrast is defined in which each variable is weighted. The weight of the variable of interest is set 1 to search for positive correlations and −1 to search for negative correlations whilst the remaining variables are not weighted (weight, 0) so that variance explained by these remaining variables (nuisance variables) are removed prior to estimation of significance. To search for the interaction of two variables, both variables as well as the interaction term (i.e. the product of both variables) are included in the GLM. Now, only the interaction term is weighted (again 1 or −1). This way, variance merely explained by the main effects will be removed so that only the interaction of the two variables is estimated. Intriguingly, an interaction with age will possibly result in the mere finding of a main effect if the age range of the subjects under investigation is unsuitable to detect this interaction. Since we had no hypothesis on the age at which such an interaction comes into play, we predefined the combined measure of the main effect of long CAG repeat and its interaction with age as the primary endpoint. As secondary endpoints, we determined the main effect of long CAG repeat and its interaction with age separately. For clarity, we will first describe the model to determine the main effect (1), then the model to determine the mere interaction with age (2), and, finally, the model to determine the primary endpoint, i.e. the combination of the first two effects. 1) The main effect of the long CAG repeat was estimated by inclusion of long CAG repeat, age, and sex; then, long CAG repeat size was weighted. 2) The interaction of the long CAG repeat with age was estimated by inclusion of long CAG repeat×age, long CAG repeat, age, and sex; then, CAG repeat×age was weighted. 3) The combined effect of 1) and 2) was estimated by inclusion of long CAG repeat×age, age, and sex (but not long CAG); then, long CAG repeat×age was weighted.

We expected changes that, compared to HD, are of relatively small effect size and located within subcortical areas since, in HD, atrophy is most pronounced here. Hence, we performed a region of interest (ROI) analysis. This single and bilateral ROI included the striatum, pallidum, and thalamus. Since structural variance explained by the long CAG repeat size in normal subjects must not necessarily be pronounced in regions, which primarily display GM loss in HD, we also performed a whole brain analysis. We applied a height threshold (voxel level) of p<0.05 corrected according to the family-wise error [9].

For exploratory analyses, we relaxed the height threshold (voxel level) to 0.01 and 0.05 uncorrected. Now, we considered the corresponding area of the opposite hemisphere and also determined P values derived from both cluster level inference (extent threshold), which gives a single p value for each observed cluster, and set level interference, which gives a single p value for all observed clusters [9]. Beforehand, data were corrected for inhomogeneity of smoothness which is necessary for cluster size analyses of VBM data in particular [10].

In retrospect (during the course of the review process), we performed the same analyses with regard to the effect of the short CAG repeat.

Location of basal ganglia structures was assessed according to the Harvard-Oxford subcortical structural atlas (http://www.fmrib.ox.ac.uk/fsl/data/atlas-descriptions.html).

## Results

Within the margins published so far [11], CAG repeat sizes ranged from 9 to 32 (mean±SD, 18.4±3.2).

**Figure 1:**
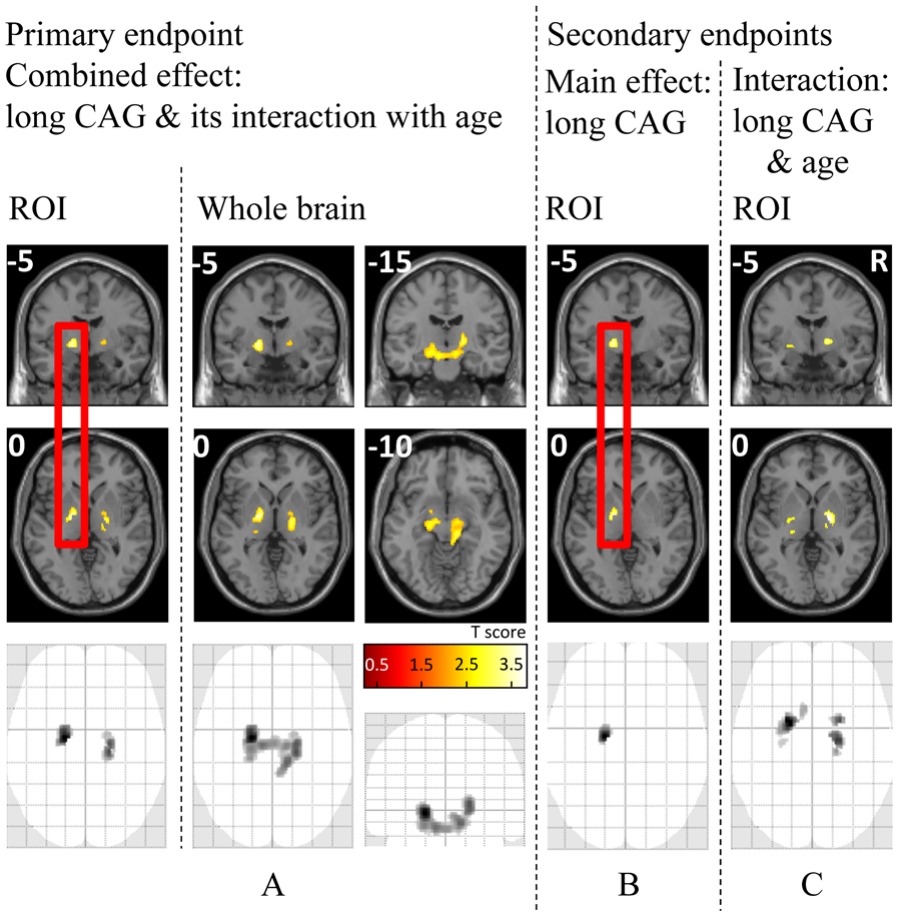
Projections of coronal (upper row) and axial (middle row) slices onto the SPM template as well as maximum intensity projections (lower row) are shown. MNI coordinates are indicated in the left upper corners. Increasing significance (T score) is color-coded from dark red to light yellow as indicated by the bar in the center. Note that only the clusters marked with a red rectangle contain peak voxels, which remained significant after correction for multiple comparisons at the voxel level. For better visibility, all results (including those from exploratory analyses) are shown at a height threshold of 0.01 uncorrected. Cluster sizes were restricted to 20 contiguous voxels for ROI analyses or subjected to cluster level correction (p<0.05 corrected) for the whole brain analysis. A) Combined effect of long CAG & its interaction with age, ROI analysis (left) and whole-brain analysis (right) revealing one bilateral cluster reaching from the pallidum across parts of the ventral thalamus to the midbrain (corrected P value at the cluster level, 0.003). B) Main effect of the long CAG, ROI analysis. C) Interaction analysis of long CAG with age, ROI analysis.

ROI analysis of the primary endpoint, i.e. the combined effect of long CAG repeat and its interaction with age, revealed a cluster of 21 voxels of increased GM with increasing long CAG repeat and its interaction with age within the left pallidum (Fig. 1A, Table 1). Exploratory whole brain analysis at the uncorrected height threshold of 0.01 revealed a large cluster of GM increase, which survived whole brain correction at the cluster level (p = 0.003) and which reached from the pallidum across parts of the ventral thalamus to the midbrain (Fig. 1A). Both the right and left hemispheric maximum were located within the pallidum. Slice by slice comparison with standard atlases of the human brain stem [12], [13] revealed bilateral overlap with the nucleus subthalamicus and substantia nigra.

**Table 1:** Influence of long CAG and age on cerebral gray matter.

Region	MNI coordinates (peak)	P values	
		voxel level (corrected for ROI)	voxel level uncorrected
**Primary endpoint: combined effect of long CAG and its interaction with age**			
(Contrast weights: long CAG×age, 1; age, 0; sex, 0)			
**Pallidum L**	−17 −8 −3	**0.007**	8.4×10^−6^
**Pallidum R**	20 −12 2	0.11	0.0003
**Secondary endpoint: main effect of long CAG**			
(Contrast weights: long CAG, 1; age, 0; sex, 0)			
**Pallidum L**	−17 −8 −3	**0.02**	4.4×10^−5^
**Pallidum R**	18 −5 −2	0.8	0.037
**Secondary endpoint: effect of interaction of long CAG and age**			
(Contrast weights: long CAG×age, 1; CAG, 0; age, 0; sex, 0)			
**Pallidum L**	−20 0 −6	0.2*	0.0005
**Pallidum R**	21 −11 0	0.08*	0.0001

**Note.** L, left; R, right;

*corrected P value at the set level, 0.023.

ROI analysis of the main effect of long CAG (secondary endpoint) yielded 3 contiguous voxels of significant GM increase with increasing long CAG repeat within the left pallidum (Fig. 1B, Table 1). Relaxing the significance threshold to 0.05 uncorrected suggested GM increase also within the right pallidum (p = 0.037 uncorrected, not shown).

ROI analysis of the interaction of long CAG with age (secondary endpoint) showed no significant results according to defined significance thresholds. However, at the uncorrected height threshold of 0.01, we observed small clusters of increasing GM with increasing interaction that resulted in a corrected P value of 0.023 at the set level (Fig. 1C, Table 1). Further, decreasing the height threshold to 0.05 yielded a cluster, which overlapped with the peak voxel derived from the analysis of the main effect of long CAG.

None of the GM increases detected survived whole brain correction for multiple statistical tests at the voxel level. We did not observe any GM decrease. Subsequent analyses of the effect of the short CAG repeat did not yield any significant result.

## Discussion

We investigated the effect of the CAG repeat length in normal subjects and demonstrated an increase of GM within the left pallidum as the long CAG repeat and its interaction with age increased. In this way, we showed that the variability of the CAG repeat length within the normal range influences brain structure in normal humans. We first reason why we focused on the long CAG repeat and its interaction with age; next, we critically discuss methodological issues. Finally, we consider possible implications for the understanding of HD, although variation within other trinucleotide repeats may also influence human brain structure.

Before data analysis, we reasoned that, in analogy to HD, the long CAG is primarily efficacious with regard to brain structure. Besides, in our draw of images, the short CAG repeat size correlated with age by chance (Pearson's coefficient, −0.14; 2-sided P value, 0.019) so that it seemed hardly possible to disentangle the effect of the short CAG from that of age. Further, we did not focus on the interaction of the short and long CAG repeat length since, again in analogy to HD, we expected it to be less efficacious [14], [15], [16], [17]. Of note, short and long CAG repeat correlated significantly (Pearson's coefficient, 0.33; 2-sided P value, <0.001) which is well conceivable as only a large long CAG repeat implies the possibility of a “large” short CAG repeat (since the latter would otherwise constitute the long CAG repeat). This dependence also implied a significant correlation of the long CAG repeat with the difference from the long and short CAG repeat (Pearson's coefficient, 0.82; 2-sided P value, <0.001) so that our data are inappropriate to study differential effects of the two CAG repeat lengths and their interaction. In contrast, age and long CAG repeat size were not correlated (Pearson's coefficient, 0.013; 2-sided P value, 0.8) but almost orthogonal so that our data were suitable to analyze the interaction of long CAG repeat size and age. Our assumption on this interaction was not only based on the fact that HD is a neurodegenerative and, hence, progressive disease but also on knowledge about normal Huntingtin whose increased expression leads to protection from apoptotic neuronal cell death after toxic stimuli, neuroprotection from excitotoxicity, and increased transcription of brain-derived neurotrophic factor [18]—all characteristics that may well interfere with the process of aging. Yet our detailed analyses indicated that the interaction of long CAG repeat size with age was minor compared to the main effect of the long CAG repeat (i.e. an increase of GM with increasing long CAG) which may result from the age distribution of our population that was relatively young (75%, <41 years).

Statistical analyses of MRI data throughout the whole brain are conservative given the need to correct for multiple statistical tests. Consequently, ROI analyses are commonly applied. This approach is justified by empirical results from imaging genetics that found no relationship between certain ROIs and false positives [19]. Still ROI analyses are only acceptable if this particular region was predicted in advance [9]. Thus, ROI selection requires critical assessment. We included the striatum since the medium spiny neurons of this basal ganglia structure are most affected in HD [2] so that we expected GM changes to be most striking here. We also included the pallidum and thalamus since medium spiny neurons mainly project to the pallidum and, from there, to the thalamus [20]. Accordingly, disturbance of the indirect and direct pathway is commonly assumed in HD, and pronounced GM loss within the pallidum and thalamus has been described [21], [22]. Of note, evidence exists that even points to an influence of the normal CAG repeat size on pallidum structure in HD. Aziz et. al. investigated HD patients with regard to the influence of the normal CAG repeat size (i.e., the CAG repeat size of the chromosome homologous to the mutant chromosome) on the course of the disorder and demonstrated an interaction of the expanded (mutant) CAG repeat with its normal counterpart [14] although others could not completely replicate this finding [16]. Preliminary data of MRI scans from 16 patients even indicated a main effect of the normal CAG repeat on basal ganglia structures (i.e. a linear negative relationship between normal CAG repeat and pallidum volume) which was most pronounced within the pallidum [14]. In our primary endpoint analysis however, only parts of the left pallidum survived correction for multiple statistical tests. On the other hand, analyses of the secondary endpoints yielded plausible results supporting our main finding. Relaxing the statistical significance threshold suggested a main effect of long CAG also within the right pallidum (i.e. an increase of GM with increasing long CAG). Moreover, interaction analysis of long CAG with age yielded clusters overlapping with those of the main effect analysis resulting in a set level P value of 0.023 although the result of this interaction analysis is independent from that of the main effect analysis. Further, exploratory whole brain analysis of the primary endpoint, the combined effect of the long CAG repeat and its interaction with age, at the height threshold of 0.01 (Fig. 1C) yielded a single cluster, which survived whole-brain correction (cluster level), largely overlapped with our ROI and, thus, with regions critically involved in HD. Besides the pallidum, we detected parts of the ventral thalamus (station of the direct and indirect pathway) as well as parts of the mesencephalon (overlapping with the nucleus subthalamicus, a station of the indirect pathway, and substantia nigra which exerts a modulatory effect on both the direct and indirect pathway). Finally, “conventional” structural T1-weighted MRIs, as used here, may not provide sufficient contrast for reliable automated segmentation accuracy of subcortical structures including the pallidum [23] although others could identify GM changes within the pallidum in asymptomatic heterozygous Parkin mutation carriers [24]. Of note, our segmentation algorithm accounts for partial volume effects [5]. Yet we considered the mean GM value of our peak voxel (MNI coordinates −17 −8 3) in retrospect. This calculation yielded a value of 0.3±0.0084 (mean±SD) indicating a sufficient amount of GM detected by the methods applied here. Still, we acknowledge as a limitation of our methodology that GM segmentation within the pallidum was not as accurate as for most cortical regions and the striatum where values ranged around 0.8.

Next, we will briefly review on Huntingtin, the gene product of *IT15* and consider possible implications of our finding for the understanding of HD [25]. Given that Huntingtin is expressed across and outside the brain and that neither the physiological nor pathological role is fully understood, we are far from a unifying model of HD's pathophysiology. Nevertheless, the understanding of normal Huntingtin's function has been regarded an important approach to HD because of several experimental findings: increased expression of normal Huntingtin improves brain cell survival; removal of normal Huntingtin generates some of the phenotypes also observed in the presence of mutant Huntingtin; normal Huntingtin expression mitigates the effect of the mutant protein; and deletion of the normal allele in an animal model of HD causes more damage [18]. The function of Huntingtin at the molecular level is less clear however. It is a soluble protein of 3,144 amino acids with many potential domains. CAG repeats are translated into the polyglutamine tract (polyQ) near the N-terminal. This portion forms a polar zipper [26] suggesting a physiological function to bind—assumingly numerous—transcription factors that also contain a polyQ region. Differences in CAG repeat size and, hence, the polyQ region may alter this binding through conformational changes, which is a testable hypothesis. Within the normal range, subtle structural changes may result whilst, in HD, the profile of respective binding partners may be altered dramatically leading to cytotoxicity. On the other hand, it also seems possible that normal CAG repeat size influences functions that facilitate or mitigate HD symptoms at the systemic level. Since longitudinal VBM studies demonstrated that an increased signal goes along with increased functional capacity [27], [28], our finding of increased GM within structures attributable to the indirect pathway may imply a predisposition to hyperkinesia. In HD, mild to moderate CAG increase goes along with hyperkinesia and later onset. Hence, long normal CAG repeat size could aggravate symptoms in these patients. In contrast, high mutant CAG repeat size results in hypokinesia and earlier onset so that high normal CAG repeat size could antagonize and, hence, mitigate symptoms. Of note, latest evidence of an effect of the normal CAG repeat size on the course of HD points in the same direction. A long normal CAG size increased pathogenicity (i.e. earlier onset of symptoms) in patients with an expansion of <44 CAG repeats whilst a long normal CAG repeat size exerted a protective effect in patients with an expansion of >44 CAG repeats (i.e. later onset of symptoms) [14]. These considerations raise the question whether normal CAG repeat size plays a role for any other phenotype than HD? Such evidence does not yet exist however. Schizophrenia [29], affective disorders [30], [31], and Parkinson's disease [32] could be related to neither increased nor decreased CAG repeat size.

In summary, we demonstrated that CAG repeat length within *IT15* influences normal brain structure in humans. This finding may help to understand variation of human brain structure in both health and disease.
